# Mandibular Atrophy and Its Impact on Overdenture Performance: Insights From a 5‐Year Longitudinal Study

**DOI:** 10.1111/joor.70113

**Published:** 2025-11-26

**Authors:** Fernanda Faot, Luciana Rezende Pinto, Lucas Jardim Da Silva, Laura Lourenço Morel, Otacílio Luiz Chagas‐Júnior, Anna Paula Da Rosa Possebon

**Affiliations:** ^1^ Department of Restorative Dentistry, School of Dentistry Federal University of Pelotas (UFPel) Pelotas RS Brazil; ^2^ Graduate Program in Dentistry, School of Dentistry Federal University of Pelotas (UFPel) Pelotas RS Brazil; ^3^ Department of Oral and Maxillofacial Surgery and Maxillofacial Prosthodontics, School of Dentistry Federal University of Pelotas (UFPel) Pelotas RS Brazil

**Keywords:** atrophic mandible, implant‐retained mandibular overdentures, prosthetic maintenance, quality of life

## Abstract

**Background:**

The long‐term influence of mandibular atrophy on masticatory function, patient‐reported outcomes, and prosthetic maintenance in users of implant‐retained overdentures remains unclear.

**Objective:**

To assess the impact of mandibular bone atrophy on masticatory function, quality of life, patient satisfaction, and prosthetic maintenance over a five‐year period.

**Methods:**

Twenty‐four fully edentulous patients were rehabilitated with mandibular overdentures retained by two narrow‐diameter implants and divided into two groups according to mandibular bone height: atrophic mandible (AM) and non‐atrophic mandible (NAM). Masticatory performance (MP) and swallowing threshold (ST) were assessed, while quality of life and satisfaction were evaluated using the Dental Impact on Daily Living (DIDL) questionnaire. Prosthetic maintenance events were recorded over 5 years. Multilevel mixed‐effects regression evaluated temporal trends and group differences, and chi‐square tests were used for prosthetic events.

**Results:**

Four participants were lost to follow‐up, resulting in 10 individuals per group at 5 years. The AM group showed significant reductions in MP_X50 (–9.66%; *p* = 0.00) and ST_X50 (–1.9%; *p* = 0.01); and increase in ST_ME5.6 (+43.32%; *p* = 0.01). The eating/chewing domain of the DIDL was significantly lower in the AM group (0.35 ± 0.72) compared to the NAM group (0.73 ± 0.47). Although overall prosthetic maintenance did not differ significantly, the AM group exhibited a higher frequency of Equator dislodgement (9.24%; *p* = 0.00) and attachment replacement (6.02%; *p* = 0.00).

**Conclusion:**

Mandibular atrophy adversely affected masticatory function, patient‐reported chewing satisfaction, and the frequency of specific prosthetic complications over 5 years.

## Graphical abstract

Overview of the 5‐year follow‐up evaluating how mandibular atrophy influences masticatory function, OHRQoL, satisfaction, and prosthetic maintenance in implant‐retained mandibular overdentures, with objective, sample distribution, methodology, outcomes, and clinical recommendations.

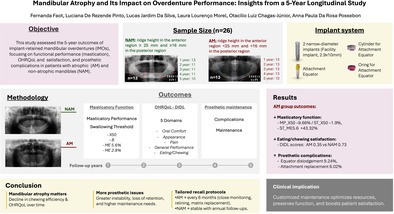

## 1 Introduction

The positive impact of rehabilitating edentulous patients with mandibular overdentures has been extensively documented over the past 10 years [1, 2, 3]. Although numerous outcomes consolidate the benefits provided to the well‐being of edentulous patients, most studies focus on the predictability of implant systems [4] and various restorative biomechanical options [5, 6, 7, 8, 9]. However, a significant number of edentulous patients remain hesitant to adopt implant‐retained prostheses, and their willingness to pay for this type of treatment shows that it remains out of reach for a large segment of this population [10, 11]. Consequently, the patients who are more likely to adhere to and invest in this treatment approach are those who have severe mandibular ridge atrophy due to prolonged edentulism and have had multiple negative experiences related to conventional complete dentures (CDs) retention and stability [12, 13, 14].

Few studies differentiate and explore functional behaviour [15, 16, 17, 18, 19] or the user perception among individuals with different levels of bone atrophy [20, 21, 22, 23]. Kimoto and Garrett [15] evaluated the effects of three levels of bone atrophy, based on the height of the mandibular symphysis (low, ≤ 21 mm; moderate, > 21 mm < 28 mm; and high, ≥ 28 mm), on the masticatory function of patients using CDs and implant‐retained mandibular overdentures (IMOs). They applied a swallowing threshold test using peanuts and carrots after 6 months of use. The results showed significant average differences between CDs and IMOs for both types of food only in the low bone height group. This suggests that only patients with advanced ridge resorption are more likely to experience improvements in masticatory performance with IMOs.

In contrast, a previous 1‐year longitudinal study observed differences in the masticatory function of atrophic and non‐atrophic patients at the third month after the conventional loading of 2‐implant overdentures [17]. During this short transition period, patients with atrophic mandibles showed less particle homogeneity when chewing an artificial test food (Optocal) in the swallowing threshold test. However, these differences disappeared after 6 and 12 months. After 3 years of using IMOs, this same sample showed no significant differences between individuals with and without ridge atrophy regarding masticatory function parameters, except for a transient 32% increase in the number of cycles required to complete the swallowing threshold test in non‐atrophic patients [19].

Implant‐retained mandibular overdentures substantially reduce the problems that lead to functional limitations, consequently improving masticatory function and OHRoL. However, patients who still experience unsatisfactory masticatory function after transitioning to implant‐retained mandibular overdentures face more psychological discomfort [24]. In addition, over time, a reduction in masticatory function can lead to difficulties chewing a variety of foods, dietary restrictions, and potential nutritional deficiencies. The psychological impact of these limitations can be substantial, contributing to self‐consciousness, social withdrawal, and decreased overall life satisfaction. Discomfort and inconvenience from poorly fitting mandibular overdentures further highlight the importance of regular maintenance and adjustments. Despite all these problems raised, medium‐term results from a clinical study showed that no problems or complaints were reported related to satisfaction or quality of life irrespective of mandible atrophy in IMO users till 3 years of usage [19].

To maintain the quality of prostheses over time, maintenance and repairs are commonly required due to the natural wear of prosthetic materials and intrinsic patient factors such as habits, masticatory force, oral hygiene, diet, and food composition. Therefore, it is essential for patients to return regularly to the dentist for evaluations and, if necessary, perform maintenance or repairs. The maintenance regime of IMOs plays a critical role in influencing both mastication and quality of life over time [23, 25, 26]. Regular check‐ups and adjustments are essential to ensure the continued fit and function of the overdenture, addressing issues such as wear and tear of prosthetic components, changes in oral tissues, and implant stability. Proactive maintenance can help mitigate the effects of bone resorption and mandibular atrophy, ensuring that overdentures remain stable and effective for mastication. Additionally, prosthetic complications may arise over the years, particularly in patients with varying degrees of alveolar ridge resorption, as atrophy is associated with a higher incidence of such complications [19, 23].

Despite the benefits of IMOs, there is evidence indicating that overdenture wearers experience a decline in masticatory function over time, incipiently observed in some masticatory outcomes between 3 and 5 years of use. This decline can be attributed to various factors, including the condition of the supporting structures, mandibular atrophy, neuromuscular adaptations required for effective use, aging, and the mechanical properties of the overdentures. The timeline of this decline is crucial for understanding clinical management and intervention strategies to mitigate its effects. However, longitudinal studies aiming to investigate and determine the long‐term functionality of IMOs are still scarce in the literature.

The aim of this study was to investigate the evolution of functional parameters such as masticatory function, satisfaction and OHRQoL in a sample of IMO users with atrophic (AM) and non‐atrophic mandibles (NAM) previously evaluated [17, 19] as well as monitoring the prosthetic complications in each group over 5 years of follow‐up after IMO rehabilitation. The null hypothesis of the present study is that no significant differences will be observed between the groups for any evaluated outcome throughout the 5‐year follow‐up.

## 2 Methodology

The data presented are part of a 5‐year follow‐up clinical study approved by the local Research Ethics Committee (69/2013 and no. 3.725.829) of the Federal University of Pelotas (UFPel). Totally edentulous patients rehabilitated with conventional CDs in both arches were previously categorized into two groups according to their level of bone atrophy based on the Cawood and Howel [27] classification: (i) AM—patients with anterior bone height less than 25 mm and posterior bone height less than 16 mm; and (ii) NAM—patients with anterior and posterior bone heights equal to or greater than 25 mm and 16 mm, respectively. After the placement of 2 narrow‐diameter implants (Ø2.9 × 10 mm; Facility; Neodent) retained by button‐type attachments (Equator Attachment; Neodent), the mandibular CCDs were converted into IMOs, and participants were evaluated annually over 5 years. The sample size calculation was redone for this 5‐year follow‐up, using a previous 1‐year study [21], with the outcome X50 (Time 1: mean 5.29, SD 1.15; Time 2: mean 3.17, SD 1.37). Thus, six individuals per group were required, totaling 12. With a 20% increase to avoid losses, a total of 15 individuals were needed to detect differences in masticatory function.

The questionnaire used to measure OHRQoL was the Dental Impact on Daily Living (DIDL), which has 36 questions divided into five domains: Oral Comfort, Appearance, Pain, General Performance, and Eating/Chewing. Additionally, the score for each domain allows classification of patients as satisfied (0.7–1.0), relatively satisfied (0–0.69), or dissatisfied (< 0) [28]. Effect sizes were interpreted as follows: small (0.2 ≤ ES < 0.5), moderate (0.5 ≤ ES < 0.8), and large (ES ≥ 0.8).

Regarding prosthetic maintenance, these data included prosthetic complications related to the attachment system and to the prostheses (CDs and IMOs), such as: Equator dislodgement, matrix/female dislodgement, prosthetic fractures, new dentures, Equator changes, matrix recaptures, teeth fractures, matrix change, reopening for abutment replacement, removal of keratinized peri‐implant mucosa, and prosthesis rebasing or adjustment, and nylon O‐ring replacement. The type of complication, number of events, and percentages were recorded.

Masticatory function was evaluated through masticatory performance (MP, 40 masticatory cycles) and the Swallowing Threshold (ST, free number of masticatory cycles) by asking participants to chew a pre‐weighed portion (3.7 g) of an artificial test food. After chewing, the material was expectorated onto filter paper, dried at room temperature for 7 days, and processed by a sieve fractionation method. Nine sieves with mesh sizes ranging from 5.6 to 0.5 mm were mounted on a magnetic agitator, and the material retained on each sieve was weighed for calculation of X50 and B using the Rosin‐Rammler formula. The X50 value corresponds to the theoretical aperture of the sieve through which 50% of the crushed particles pass, and the B index describes the homogeneity of the particles [29]. To complement the analysis of masticatory function, masticatory efficiency (ME_5.6 and ME_2.8), which reflects the percentages of material retained on sieves with apertures of 5.6 and 2.8 mm, as well as the number and duration of cycles, was also recorded.

Considering that the data had a hierarchical structure, as time periods were nested within participants, a multilevel mixed‐effects regression was performed to assess trends in changes in the DIDL domain scores over the years for each group. For the presentation of results, a multilevel mixed‐effects regression was conducted to evaluate trends in changes in OHRQoL and masticatory function over time in each group and to investigate differences between the groups, with the NAM group adopted as the reference at the end of the 5th year. For differences between groups in relation to maintenance, a chi‐square test was performed. All tests were conducted using Stata 14.1 (StataCorp., College Station, TX, USA).

## 3 Results

A total of 24 patients from a previous study [19] were invited to undergo the 5‐year evaluation of IOM use. At baseline, mandibular bone height was greater in the NAM group at both the anterior midline (26.1 ± 5.3 mm) and molar region (17.4 ± 4.1 mm) compared with the AM group (20.6 ± 2.8 mm and 11.9 ± 2.4 mm). After 5 years, reductions were observed in both groups, with the NAM group showing greater anterior reduction (−4.4 mm vs. −3.0 mm), while molar height decreased similarly (−1.5 mm in both groups). Importantly, no participant initially classified as NAM reached the atrophy threshold, indicating that the group allocation remained stable throughout follow‐up.

At the 5th year, four losses to follow‐up occurred (3 in the AM group: two due to death and one due to not returning to consultations because of the COVID‐19 pandemic; and one in the NAM group—death), leaving 10 individuals in each group. In the AM group, seven were women, and in the NAM group, four were men. The mean age was 70.4 years in the AM group and 68.2 years in the NAM group.

Differences in masticatory function between the groups (Table 1) at the end of 5 years of IMO use were significant for the following outcomes: MPX50 (*p* = 0.00), STX50 (*p* = 0.01), and ST_ME5.6 (*p* = 0.01). For all these outcomes, the AM group showed worse values than the NAM group (Table S1), grinding 9.66% fewer particles in the MPX50 test. In the ST test, there was a 43.32% higher retention of particles on the 5.6 mm sieve, resulting in a slight 1.9% worsening in particle grinding (STX50).

**TABLE 1:** Regression of DIDL and outcomes related to masticatory function between non‐atrophic (NAM) and atrophic (AM) individuals at 5 years (mixed‐effects multilevel linear regression).

	NAM	AM
	Ref.	Coefficient (*p*) = confidence interval
DIDL domains		
Appearance	1.00	0.14 (0.48) = −0.25; 0.53
Pain	1.00	0.04 (0.87) = −0.48; 0.56
Oral comfort	1.00	−0.05 (0.81) = −0.49; 0.38
General performance	1.00	0.06 (0.48) = −0.12;0.25
Eating/chewing	1.00	**0.34 (0.03) = 0.02; 0.65**
Masticatory performance		
X50	1.00	**−0.44 (0.00) = −0.76; −0.11**
B	1.00	−0.12 (0.22) = −0.32; 0.07
ME 5.6	1.00	−0.37 (0.06) = −0.78‐;0.02
ME 2.8	1.00	−0.40 (0.12) = −0.91; 0.10
Swallowing threshold		
X50	1.00	**−0.36 (0.01) = −0.66; −0.07**
B	1.00	−0.30 (0.28) = −0.87; 0.25
Time	1.00	0.28 (0.38) = −0.35; 0.92
Cycles	1.00	−0.09 (0.75) = −0.65; 0.47
ME 5.6	1.00	**−0.45 (0.01) = −0.82; −0.09**
ME 2.8	1.00	0.19 (0.31) = −0.18; 0.57

*Note:* Bold values indicate statistical significance at *p* < 0.05.

However, exclusively for the AM group (Table 2 and Table S1), changes in masticatory outcomes were observed over the years, mainly for MPX50 between the 5th year and all previous years (5 × 4, *p* = 0.00; 5 × 3, *p* = 0.01; 5 × 2, *p* = 0.00 and 5 × 1, *p* = 0.00). For MPB, there was a significant change only between 5 and 2 years (*p* = 0.02), with a 3% improvement in homogeneity. For the 5.6 mm sieve retention, changes were also observed between all evaluated periods (5 × 4, *p* = 0.01; 5 × 3, *p* = 0.00; 5 × 2, *p* = 0.00; 5 × 1, *p* = 0.02), with an increase in retention in the 5th year compared to all previous periods, the greatest increase being between 4 and 5 years (36.32%). For the 2.8 mm sieve retention, the change occurred from the 5th to the 3rd year (*p* = 0.04) and to the 1st year (*p* = 0.02), with a slight 2.89% worsening in retention between the 3rd and 5th year. Regarding ST, the time required to perform masticatory cycles increased from 54.95 to 57.86 s between 5 and 4 years (*p* = 0.00) and from 53.64 to 57.86 s between 5 and 3 years (*p* = 0.00). The number of cycles increased from 58.92 to 69.30 between 5 and 3 years (*p* = 0.00).

**TABLE 2:** Change in masticatory performance (MP) and swallowing threshold (ST) within the group over time (mixed‐effects multilevel linear regression).

Test	Group	Outcome	Time [coefficient (*p*) = confidence interval]				
			1 year	2 years	3 years	4 years	5 years
MP	NAM	MPX50	−0.23 (0.33) = −0.71; 0.21	−0.11 (0.63) = −0.60; 0.36	−0.24 (0.32) = −0.74; 0.24	−0.19 (0.37) = −0.63; 0.23	1.00
		MPB	−0.01 (0.87) = −0.23; 0.10	0.09 (0.70) = −0.39; 0.58	−0.02 (0.91) = −0.40; 0.36	−0.05 (0.66) = −0.28; 0.18	1.00
		ME 5.6	−0.37 (0.14) = −0.87; 0.13	−0.14 (0.59) = −0.68; 0.39	−0.34 (0.23) = −0.92; 0.22	−0.21 (0.45) = −0.78; 0.35	1.00
		ME 2.8	−0.09 (0.73) = −0.62; 0.44	−0.08 (0.75) = −0.59; 0.43	0.14 (0.69) = −0.57; 0.86	**0.60 (0.02) = 0.08; 1.11**	1.00
	AM	MPX50	**0.67 (0.00) = 0.23; 1.12**	**0.76 (0.00) = 0.43; 1.10**	**0.58 (0.01) = 0.10; 1.05**	**0.57 (0.00) = 0.17; 0.97**	1.00
		MPB	0.45 (0.11) = −0.10; 1.01	**0.54 (0.02) = 0.06; 1.02**	0.27 (0.47) = −0.48; 1.03	0.32 (0.47) = −0.57; 1.23	1.00
		ME 5.6	**0.58 (0.02) = 0.06; 1.11**	**0.67 (0.00) = 0.27; 1.07**	**0.74 (0.00) = 0.26; 1.21**	**0.66 (0.01) = 0.15; 1.16**	1.00
		ME 2.8	**0.48 (0.02) = 0.05; 0.90**	0.33 (0.20) = −0.18; 0.85	**0.62 (0.04) = 0.12; 1.23**	0.66 (0.06) = −0.01; 1.35	1.00
ST	NAM	STX50	0.25 (0.09) = −0.04; 0.54	**0.51 (0.02) = 0.07; 0.94**	0.06 (0.78) = −0.41; 0.54	**0.44 (0.01) = 0.10; 0.78**	1.00
		STB	−0.09 (0.41) = −0.31; 0.13	−0.19 (0.38) = −0.62; 0.23	−0.04 (0.89) = −0.71; 0.62	0.09 (0.55) = −0.22; 0.40	1.00
		Time	**0.63 (0.02) = 0.07; 1.18**	0.24 (0.61) = −0.68; 1.16	0.74 (0.06) = −0.01; 1.50	**0.89 (0.00) = 0.36; 1.43**	1.00
		Cycles	**0.94 (0.00) = 0.26; 1.62**	**1.16 (0.01) = 0.25; 2.07**	**0.48 (0.03) = 0.03; 0.93**	**0.83 (0.00) = 0.14; 1.26**	1.00
		ME 5.6	0.22 (0.09) = −0.03; 0.48	**0.49 (0.02) = 0.05; 0.94**	**0.48 (0.00) = 0.21; 0.76**	0.39 (0.06) = −0.01; 0.81	1.00
		ME 2.8	0.14 (0.53) = −0.31; 0.61	0.12 (0.45) = −0.19; 0.44	**0.41 (0.01) = 0.07; 0.74**	0.02 (0.91) = −0.45; 0.50	1.00
	AM	STX50	**0.32 (0.00) = 0.08; 0.56**	**0.30 (0.01) = 0.05; 0.56**	0.22 (0.07) = −0.02; 0.47	**0.38 (0.00) = 0.13; 0.62**	1.00
		STB	0.01 (0.92) = −0.21; 0.24	−0.11 (0.37) = −0.36; 0.13	0.02 (0.60) = −0.07; 0.12	0.05 (0.80) = −0.34; 0.44	1.00
		Time	0.11 (0.45) = −0.18; 0.41	0.34 (0.08) = −0.04; 0.72	**0.60 (0.00) = 0.44; 0.76**	**0.64 (0.00) = 0.20; 1.08**	1.00
		Cycles	−0.11 (0.63) = −0.61; 0.37	0.40 (0.13) = −0.12; 0.92	**0.49 (0.08) = 0.12; 0.86**	0.54 (0.12) = −0.14; 1.27	1.00
		ME 5.6	**0.29 (0.01) = 0.06; 0.51**	0.09 (0.57) = −0.23; 0.43	0.17 (0.15) = −0.06; 0.40	**0.40 (0.00) = 0.15; 0.66**	1.00
		ME 2.8	0.16 (0.27) = −0.13; 0.46	−0.22 (0.10) = −0.50; 0.04	**0.36 (0.01) = 0.08; 0.64**	**0.80 (0.01) = 0.15; 1.45**	1.00

*Note:* Bold values indicate statistical significance at *p* < 0.05.

For the NAM group (Table 2 and Table S1), the MP results showed a significant increase from 21.42% to 22.79% retention on the 2.8 mm sieve between 5 and 4 years (*p* = 0.02). For MPX50, MPB, and MPME 5.6, there were no significant changes between any time periods. When analysing the ST, there was an increase in the time to perform masticatory cycles from 44.32 to 51.59 s between 5 and 4 years (*p* = 0.01) and from 52.58 to 51.59 s between 5 and 1 year (*p* = 0.00), as well as a significant increase in the number of masticatory cycles at the end of 5 years. The greatest significant difference was between 5 and 2 years (from 51.27 to 59.60 cycles, *p* = 0.01).

**FIGURE 1:**
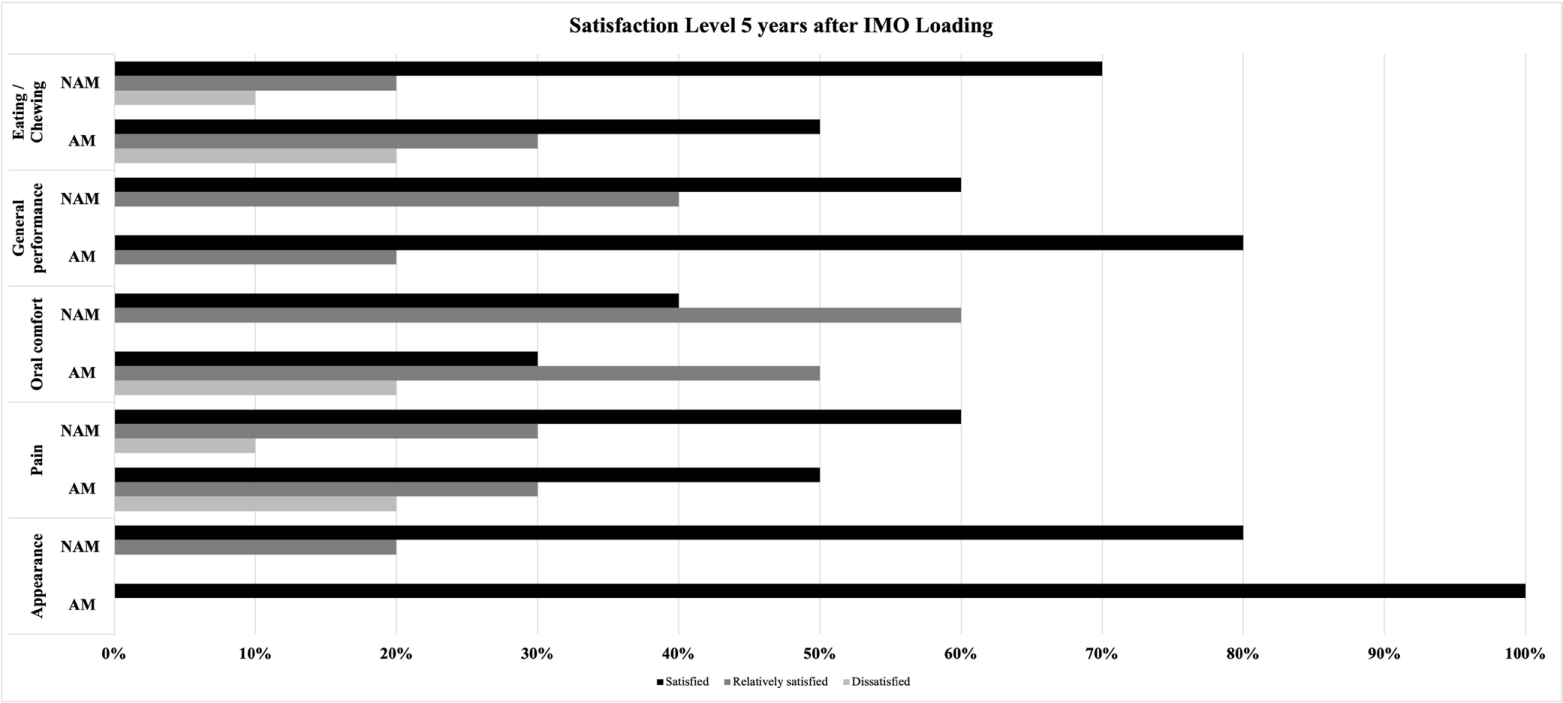
Patient satisfaction levels in each DIDL domain over 5 years: atrophic patients (AM) × non‐atrophic patients (NAM).

When analysing OHRQoL differences between groups at the 5th year (Table 1), there was a difference only in the Eating/Chewing domain (coef: 0.34; *p* = 0.03), with a lower score for the AM group (0.35 ± 0.72), showing (Table S2) that the AM group was relatively satisfied with its mastication while the NAM group was fully satisfied (0.73 ± 0.47), according to Figure 1. Examining changes in OHRQoL exclusively for the MA group over the years (Table 3), changes were found between the 5th and 4th year for the Appearance (coef: 0.97; *p* = 0.00), Pain (coef: 0.61; *p* = 0.02), Oral Comfort (coef: 0.49; *p* = 0.02), and General Performance (coef: 0.88; *p* = 0.00) domains. Additionally, for the Pain domain, there was a change between the 5th and 3rd year (coef: 2.27; *p* = 0.00), with a slight worsening in all these domain scores in the 5th year, showing that MA group participants were not fully satisfied with their rehabilitation at the end of this period in terms of Appearance, Pain, Oral Comfort, and General Performance. For the NAM group (Table 3), there was a change between the 5th and 4th year for the Appearance domain (coef: 0.46; *p* = 0.00) and the Eating/chewing domain (coef: 0.46; *p* = 0.03), with slight improvements in scores for both domains, showing that they were satisfied with denture appearance and eating/chewing at the end of 5 years. For mastication scores, there were also changes between the 5th year and all previous years (5 × 3—coef: 0.74; *p* = 0.01; 5 × 2—coef: 2.36, *p* = 0.00; 5 × 1—coef: 0.59; *p* = 0.00; 5 × 1—coef: 0.59; *p* = 0.00), with the worst scores at 5 years compared to the first years, indicating that they were more satisfied with their mastication in the first 3 years. Moreover, there were changes in the Oral Comfort domain between the 5th and 3rd year (coef: 1.97; *p* = 0.00) and between the 5th and 2nd year (coef: 1.72; *p* = 0.01), showing a decrease in satisfaction in this domain at 5 years. For General Performance, changes were observed between the 5th and 1st year (coef: 0.69; *p* = 0.04), also indicating a worse score between these two periods.

**TABLE 3:** Changes in DIDL domains over time within each group (mixed‐effects multilevel linear regression).

	1 year	2 years	3 years	4 years	5 years
	Coef. (*p*) = CI	Coef. (*p*) = CI	Coef. (*p*) = CI	Coef. (*p*) = CI	Ref.
NAM					
Appearance	**	−0.27 (0.60) = −1.32; 0.76	0.18 (0.13) = −0.05; 0.42	**0.46 (0.00) = 0.25; 0.66**	1.00
Pain	0.66 (0.22) = −0.41; 1.75	1.12 (0.07) = −0.11; 2.36	0.72 (0.38) = −0.92; 2.37	0.18 (0.66) = −0.67; 1.05	1.00
Oral comfort	0.44 (0.25) = −0.31; 1.20	**1.72 (0.01) = 0.40; 3.03**	**1.97 (0.01) = 0.78; 3.15**	0.55 (0.18) = −0.25; 1.37	1.00
General performance	**0.69 (0.04) = 0.00; 1.39**	0.71 (0.20) = −0.38; 1.81	0.04 (0.80) = −0.30; 0.39	−0.78 (0.15) = −1.86; 0.29	1.00
Eating/chewing	**0.59 (0.00) = 0.31; 0.86**	**2.36 (0.00) = 1.27; 3.45**	**0.74 (0.01) = 0.18; 1.34**	**0.46 (0.03) = 0.03; 0.88**	1.00
AM					
Appearance	**	**	−0.27 (0.52) = −1.10; 0.56	**0.97 (0.00) = 0.89; 1.05**	1.00
Pain	−0.05 (0.99) = −1.35; 1.34	**	**2.27 (0.00) = 0.84; 3.69**	**0.61 (0.02) = 0.07; 1.16**	1.00
Oral comfort	0.21 (0.69) = −0.85; 1.27	−0.24 (0.65) = −1.35; 0.83	−0.07 (0.99) = −2.60; 2.59	**0.49 (0.02) = 0.06; 0.93**	1.00
General performance	−1.23 (0.40) = −0.415; 1.68	**	1.57 (0.18) = −0.77; 3.93	**0.88 (0.00) = 0.69; 1.07**	1.00
Eating/chewing	**	**	−1.06 (0.21) = −2.73; 0.61	0.36 (0.41) = −0.51; 1.24	1.00

*Note:* ** Collinearity. Bold values indicate statistical significance at *p* < 0.05.

Regarding prosthetic maintenance and complications during the 5th year (Table 4), a total of 51 maintenance events were recorded—29 in the AM group and 22 in the NAM group—with no statistically significant difference in the total number of maintenance procedures between the groups (*p* = 0.32). However, when considering the cumulative number of prosthetic complications and maintenance events over the study period (*n* = 462), the AM group accounted for 249 events, while the NAM group recorded 213 events, demonstrating a statistically significant difference in the total number of events between the groups (*p* = 0.00). Group‐specific differences were also observed: the AM group exhibited a higher frequency of prosthetic complications such as Equator dislodgement events (9.24%; *p* = 0.00) and reopening for abutment replacement (6.02%; *p* = 0.00). In contrast, when analyzing prosthesis‐related maintenance, the NAM group showed greater demand for interventions, including prosthesis adjustment (19.72%; *p* = 0.03) and Nylon O‐ring replacement (38.02%; *p* = 0.00), when compared to the MA group.

**TABLE 4:** Chi square test results for differences in prosthetic maintenance results at the end of the 5th year (bold letters indicate significant difference).

	4 years							5 years							Overall 5 years						
	NAM			AM			*p*	NAM			AM			*p*	NAM			AM			*p*
	NP	NE	%	NP	NE	%		NP	NE	%	NP	NE	%		NE	NP	%	NE	NP	%	
Prosthetic complications																					
Equator dislodgement	0	0	0%	1	1	2.38%	0.32	0	0	0%	0	0	0%	1.00	**2**	**2**	**0.94%**	**23**	**8**	**9.24%**	**0.00**
Matrix (female) dislodgement	0	0	0%	0	0	0%	1.00	0	0	0%	0	0	0%	1.00	1	1	0.47%	1	1	0.40%	0.91
Prosthesis fracture	0	0	0%	0	0	0%	1.00	0	0	0%	0	0	0%	1.00	5	5	2.35%	2	2	0.80%	0.17
New overdenture	1	1	2.44%	0	0	0%	0.31	0	0	0%	0	0	0%	1.00	11	11	5.16%	8	8	3.21%	0.29
Equator change	**0**	**0**	**0%**	**3**	**5**	**11.90%**	**0.02**	3	3	13.64%	5	10	34.48%	0.09	14	12	6.57%	23	10	9.24%	0.29
Matrix (female) recapture	7	9	21.95%	7	9	21.42%	0.95	4	4	18.18%	1	1	3.45%	0.08	32	20	15.02%	39	23	15.66%	0.84
Teeth fracture	0	0	0%	0	0	0%	1.00	1	1	4.54%	1	1	3.45%	0.84	6	5	2.81%	7	5	2.81%	0.99
Matrix (female) change	2	4	9.76%	3	5	11.90%	0.75	0	0	0%	1	2	6.90%	0.20	9	6	4.23%	14	9	5.62%	0.49
Reopening for abutment replacement	0	0	0%	2	3	7.14%	0.08	0	0	0%	1	1	3.45%	0.37	**1**	**1**	**0.47%**	**15**	**8**	**6.02%**	**0.00**
Removal of keratinised mucosa	0	0	0%	0	0	0%	1.00	0	0	0%	0	0	0%	1.00	2	2	0.94%	8	6	3.21%	0.09
Prosthesis rebasing	1	1	2.44%	2	4	9.52%	0.17	0	0	0%	0	0	0%	1.00	7	5	3.29%	15	12	6.02%	0.16
Prosthetic maintenance																					
Prosthesis adjustment	3	5	12.20%	1	3	7.14%	0.43	2	2	9.10%	1	1	3.45%	0.39	**42**	**13**	**19.72%**	**31**	**20**	**12.45%**	**0.03**
Nylon O‐ring replacement	**7**	**21**	**51.22%**	**6**	**12**	**28.57%**	**0.03**	5	12	54.54%	6	13	44.83%	0.49	**81**	**29**	**38.02%**	**63**	**32**	**25.30%**	**0.00**
Total	21	41	100%	25	42	100%	0.91	15	22	100%	16	29	100%	0.32	**213**		**100%**	**249**		**100%**	**0.00**

## 4 Discussion

This investigation represents the first 5‐year longitudinal clinical study to systematically evaluate the influence of mandibular atrophy on masticatory function, OHRQoL, and prosthetic maintenance in IMO users. The limited evidence available beyond short‐ and medium‐term follow‐up underscores the novelty and clinical relevance of our findings. After 5 years, participants with AM exhibited a measurable deterioration in functional performance, characterized by reduced particle comminution efficiency, lower self‐reported satisfaction with mastication, and an increased incidence of prosthetic complications. A key contribution of this study is distinguishing between absolute intergroup differences at the 5‐year mark and the divergent longitudinal trajectories observed throughout follow‐up.

Patients with AM initiated treatment from a structurally disadvantaged condition and showed a steeper decline in both functional satisfaction and prosthetic stability, whereas NAM patients maintained relatively stable outcomes, with only minor variations in satisfaction and fewer technical issues. These trends indicate that baseline mandibular morphology is a determinant of long‐term functional resilience. Clinically, this suggests that AM patients require intensive maintenance and potentially reinforced attachment strategies, while NAM patients may benefit from preventive adjustments and regular replacement of retentive components. Overall, the early functional and OHRQoL improvements consistently documented in IMO users [17, 21, 24, 30] are not uniformly sustained in the long term, particularly among individuals with advanced resorption in the mandible.

The 9.66% reduction in particle grinding efficiency (MPX50) and 43.32% increase in particle retention on the 5.6 mm sieve (ST_ME5.6) that resulted in worsening in particle grinding also during ST test (STX50), confirms that low mandibular bone availability compromises masticatory function over time. A previous 3‐year study [19] reported no difference between AM and NAM individuals; however our findings demonstrate that this apparent equalisation does not persist after 5 years. These results emphasise the importance of long‐term evaluation. Similar deterioration has been reported in patients with severe maxillary bone loss (Combination Syndrome), with reduced bolus homogenization in both tests, MP and ST tests; prolonged masticatory cycles; and inferior performance in the ST [31].

Regarding masticatory function according to each group, the AM group exhibited variability in MP values that stabilized around the fourth year, followed by a slight decline in the fifth, particularly reflected in the higher percentage of larger particles retained on the 5.6 mm sieve (ME 5.6). This suggests that, despite rehabilitation, individuals with reduced mandibular height continue to experience challenges in maintaining efficient food comminution. In contrast, the NAM group maintained consistent MP values with smaller variations, indicating that greater bone support contributes to more stable masticatory efficiency. Importantly, in both groups, the efficiency gains achieved in the early years were not static but continue to evolve, reflecting ongoing functional adaptation over time. The ST results reinforced these findings. The AM group required longer chewing times and a greater number of cycles at 5 years, indicating compensatory mechanisms to overcome limited comminution. Although such efforts did not always translate into improved masticatory efficiency, they reflect adaptation rather than decline. The NAM group showed steadier performance, but also a gradual increase in effort suggesting that adaptation is inherent to long‐term IMO use. These findings align with Schuster et al. [19], who observed transient variations in masticatory cycles that later stabilized, indicating that adaptation can depend on multiple factors, including bone condition and prosthesis stability. It is known that individuals with low bone availability, who have resorbed and atrophic mandibles, may need a longer adaptation period with IMOs than non‐atrophic individuals.

This adaptation likely relates to the posterior region of the mandible, which serves as the main support area for IMOs and may contribute to denture instability due to limited bone availability. Such reduced stability helps explain our functional findings. Although some studies [32, 33] have reported that IMO use in individuals with mandibular atrophy may stabilise or even reverse bone resorption [34], thereby contributing to effective long‐term mastication, our study demonstrated a gradual decline in some masticatory function outcomes over time. Furthermore previous studies [35, 36] have shown an increased time and number of masticatory cycles do not necessarily improve comminution, consistent with our findings that AM individuals require more time and cycles but still exhibited reduced particle breakdown at 5 years.

Objective functional changes were mirrored by patient‐reported outcomes. In the AM group, the DIDL domains related to oral comfort, pain, and eating/chewing declined significantly by year five, reflecting clinically meaningful deterioration consistent with MP and ST results. In contrast, NAM participants maintained higher satisfaction scores across most domains, with only minor declines in eating/chewing and general performance. Appearance remained stable in both groups, suggesting that aesthetic perception is less affected by mandibular morphology than function. Comparing OHRQoL outcomes across studies remains challenging, as most investigations include only shorter follow‐ups (1–2 years) [17, 18, 24]. A 3‐year study [19] using the DIDL questionnaire reported no domain differences in contrast to our results. Another 3‐year study [37], applying the OHIP‐Edent instrument found worsening functional limitation scores in participants with mandibular atrophy, suggesting reduced masticatory capacity consistent with the decline observed in our 5‐year outcomes. This deterioration may be related to the greater prosthesis volume and instability frequently seen in IMOs for patients with atrophic mandibles, as discussed by Kutkut et al. [38]. Conversely, other 5‐year studies [39, 40], have shown sustained improvements in OHRQoL and overall satisfaction, irrespective of mandibular atrophy severity.

At 5 years, the total number of prosthetic maintenance events did not differ significantly between groups. However, the cumulative number of prosthetic complications and maintenance procedures was higher in AM patients, mainly due to Equator dislodgement and mucosal reopening for abutment replacement. Differently, NAM participants required more frequent prostheses adjustments and more frequent nylon o'ring replacements. These patterns may be explained by the biomechanical behaviour of each mandibular condition. Reduced bone support in AM patients, both in height and thickness, may lead to increased movement of the IMO, thereby elevating mechanical stress in the implant region due to reduced mucosal resilience. This may result in overloading the mucosa and peri‐implant bone tissue, as well as micro‐movements of the prosthetic component connected to the implant, potentially causing failures such as dislodgement and the need for mucosal reentry for replacement [15, 38, 41]. In contrast, in NAM patients enhanced stability may concentrate mechanical stress on stud abutments during the masticatory function as these patients recovered an effective mastication, leading to more frequent nylon o'ring replacements and an increased need for prosthetic adjustments to maintain long‐term comfort and function. Evidence on long‐term IMO maintenance remains limited [42], but retention system performance depends on several factors including implant connection, shape, material properties, nylon o'rings resistance/retention force and capacity to tolerate angular discrepancies related to the implant positioning. The stud abutment used in this clinical study was the first one made available in Brazil; however, its shape and mechanical retention capacity are similar to those of other brands worldwide.

Although group allocation was defined at baseline, some initially NAM individuals may experience localized bone height reduction over time, reinforcing the need for continuous monitoring. Overall, IMO rehabilitation provides sustained benefits, yet AM patients require individualized follow‐up, including shorter recall intervals (every 6 months), with close assessment of IMO stability and timely interventions such as relining or matrix replacement. In contrast, NAM patients generally maintain stable results with annual evaluations. Tailored protocols optimize clinical resources but also enhance long‐term function and satisfaction in IMO rehabilitation.

## 5 Conclusion

After 5 years of IMO use, the degree of mandibular atrophy continues to influence masticatory function, with a decline in particle grinding observed in the AM group. Additionally, OHRQoL evaluated over time also showed a slight deterioration, especially in the Eating/Chewing domain, highlighting the need for shorter follow‐up intervals between IMO maintenance appointments. This may have occurred due to the higher number of prosthetic complications in this group, resulting from increased instability and reduced retention of IMOs. Thus, the presence of mandibular atrophy continues to exert a negative influence on masticatory function, OHRQoL, and prosthetic maintenance outcomes.

## Author Contributions

**Fernanda Faot:** conceptualization; investigation; funding acquisition; writing – review and editing; methodology; formal analysis; project administration; resources; supervision. **Luciana Rezende Pinto:** resources; formal analysis; writing – review and editing; methodology; investigation. **Lucas Jardim Da Silva:** investigation; writing – original draft; visualisation. **Laura Lourenço Morel:** investigation; formal analysis; writing – original draft; validation; visualisation; methodology. **Otacílio Luiz Chagas‐Júnior:** conceptualization; investigation; writing – review and editing; methodology; formal analysis; resources. **Anna Paula da Rosa Possebon:** investigation; methodology; validation; visualisation; formal analysis; writing – review and editing.

## Conflicts of Interest

The authors declare no conflicts of interest.

## Supporting information


**Table S1:** Mean ± standard deviation and median (confidence interval) of masticatory function results from Masticatory Performance (MP) and Swallowing Threshold (ST) tests (X50, B, ME 5.6 e ME2.8) according to atrophic mandible (AM) and non‐atrophic mandible (NAM) group.
**Table S2:** Mean ± standard deviation and median (confidence interval) of the DIDL domains of subjects with atrophic (AM) and non‐atrophic mandibles (NAM) at different evaluation periods. The effect sizes (ES) of the DIDL domains at 5 years correspond to the comparison in relation to year 1.

## Data Availability

The data that support the findings of this study are available on request from the corresponding author. The data are not publicly available due to privacy or ethical restrictions.

## References

[1] S. Abou‐Ayash , M. Fonseca , S. Pieralli , and D. R. Reissmann , “Treatment Effect of Implant‐Supported Fixed Complete Dentures and Implant Overdentures on Patient‐Reported Outcomes: A Systematic Review and Meta‐Analysis,” Clinical Oral Implants Research 34, no. S26 (2023): 177–195, 10.1111/clr.14065. 37750530

[2] M. Schimmel , M. Araujo , S. Abou‐Ayash , et al., “Group 4 ITI Consensus Report: Patient Benefits Following Implant Treatment in Partially and Fully Edentulous Patients,” Clinical Oral Implants Research 34, no. S26 (2023): 257–265, 10.1111/clr.14145. 37750516

[3] E. Velasco‐Ortega , N. Matos‐Garrido , A. Jiménez‐Guerra , et al., “Early Loading of Two Implants Supporting Mandibular Overdentures in Geriatric Edentulous Patients: A 12‐Year Follow‐Up Study,” Journal of Clinical Medicine 12, no. 11 (2023): 3825, 10.3390/jcm12113825. 37298020 PMC10253364

[4] J. Park , S. Shin , and J. Lee , “Narrow‐Diameter Versus Regular‐Diameter Dental Implants for Mandibular Overdentures: A Systematic Review and Meta‐Analysis,” Journal of Prosthodontics 32, no. 8 (2023): 669–678, 10.1111/jopr.13726. 37365991

[5] W. Liu , H. Cai , J. Zhang , J. Wang , and L. Sui , “Effects of Immediate and Delayed Loading Protocols on Marginal Bone Loss Around Implants in Unsplinted Mandibular Implant‐Retained Overdentures: A Systematic Review and Meta‐Analysis,” BMC Oral Health 21, no. 1 (2021): 122, 10.1186/s12903-021-01486-3. 33731092 PMC7968211

[6] Y. Liu , F. He , Y. Zhao , et al., “Immediate Versus Non‐Immediate Loading Protocols for Reduced‐Diameter Implants Supporting Overdentures: A Systematic Review and Meta‐Analysis,” International Journal of Oral & Maxillofacial Implants 39, no. 3 (2024): 657–664, 10.11607/jomi.10625. 38498788

[7] A. L. G. Girundi , M. C. O. Ribeiro , V. F. Vargas‐Moreno , et al., “Patient‐Reported Outcome Measures and Clinical Performance of Implant‐Retained Mandibular Overdentures With Stud and Ball Attachments: A Systematic Review and Meta‐Analysis,” Journal of Prosthetic Dentistry 131, no. 2 (2024): 197–211, 10.1016/j.prosdent.2022.02.006. 35931572

[8] F. Mahanna , M. Elsyad , S. Mourad , and H. Abozaed , “Satisfaction and Oral Health–Related Quality of Life of Different Attachments Used for Implant‐Retained Overdentures in Subjects With Resorbed Mandibles: A Crossover Trial,” International Journal of Oral & Maxillofacial Implants 35, no. 2 (2020): 423–431, 10.11607/jomi.7869. 32142580

[9] M. A. Elsyad , A. E. Abdraboh , M. M. Denewar , and S. S. Mohamed , “Prosthetic Complications and Maintenance of Different Attachments Used to Stabilize Mandibular 2‐Implant Overdentures in Patients With Atrophied Ridges: A 5‐Year Randomized Controlled Clinical Trial,” Clinical Implant Dentistry and Related Research 24, no. 4 (2022): 497–509, 10.1111/cid.13093. 35466498

[10] E. Emami , A. Alesawy , P. de Grandmont , et al., “A Within‐Subject Clinical Trial on the Conversion of Mandibular Two‐Implant to Three‐Implant Overdenture: Patient‐Centered Outcomes and Willingness to Pay,” Clinical Oral Implants Research 30, no. 3 (2019): 218–228, 10.1111/clr.13408. 30681193

[11] S. Tada , M. Kanazawa , A. Miyayasu , et al., “Patient Preferences for Different Tooth Replacement Strategies for the Edentulous Mandible: A Willingness‐To‐Pay Analysis,” Journal of Prosthodontic Research 65, no. 4 (2021): 535–540, 10.2186/jpr.JPR_D_20_00170. 33980785

[12] G. A. Borges , M. H. R. Borges , C. Dini , R. M. Marcello‐Machado , V. A. R. Barão , and M. F. Mesquita , “Prognosis of Removable Complete Dentures Considering the Level of Mandibular Residual Ridge Resorption: A Systematic Review and Meta‐Analysis,” Clinical Oral Investigations 29, no. 6 (2025): 307, 10.1007/s00784-025-06379-1. 40394268

[13] C. R. Leles , N. P. Ferreira , A. H. Vieira , A. C. V. Campos , and E. T. Silva , “Factors Influencing Edentulous Patients' Preferences for Prosthodontic Treatment,” Journal of Oral Rehabilitation 38, no. 5 (2011): 333–339, 10.1111/j.1365-2842.2010.02158.x. 21039748

[14] A. Ribeiro , A. Verissimo , A. Medeiros , R. Cardoso , L. Melo , and A. Carreiro , “Non‐Adaptation to New Mandibular Complete Dentures: A Survival Analysis and Interpretation of the Time to Adaptation,” International Journal of Prosthodontics 36, no. 4 (2023): 402–409, 10.11607/ijp.7865. 37699180

[15] K. Kimoto and N. R. Garrett , “Effect of Mandibular Ridge Height on Masticatory Performance With Mandibular Conventional and Implant‐Assisted Overdentures,” International Journal of Oral & Maxillofacial Implants 18, no. 4 (2003): 523–530. 12939003

[16] C. Spitzl , P. Pröschel , M. Wichmann , and S. Heckmann , “Long‐Term Neuromuscular Status in Overdenture and Complete Denture Patients With Severe Mandibular Atrophy,” International Journal of Oral & Maxillofacial Implants 27, no. 1 (2012): 155–161. 22299092

[17] R. M. Marcello‐Machado , F. Faot , A. J. Schuster , A. M. Bielemann , J. O. L. Chagas , and A. A. Del Bel Cury , “How Does Mandibular Bone Atrophy Influence the Masticatory Function, OHRQoL and Satisfaction in Overdenture Wearers? Clinical Results Until 1‐Year Post‐Loading,” Journal of Oral Rehabilitation 44, no. 11 (2017): 850–859, 10.1111/joor.12546. 28741684

[18] A. P. d. R. Possebon , R. M. Marcello‐Machado , A. M. Bielemann , A. J. Schuster , L. R. Pinto , and F. Faot , “Masticatory Function of Conventional Complete Denture Wearers Changing to 2‐Implant Retained Mandibular Overdentures: Clinical Factor Influences After 1 Year of Function,” Journal of Prosthodontic Research 62, no. 4 (2018): 479–484, 10.1016/j.jpor.2018.06.002. 30006264

[19] A. J. Schuster , A. P. d. R. Possebon , R. M. Marcello‐Machado , O. L. Chagas‐Júnior , and F. Faot , “Masticatory Function and Oral Health‐Related Quality of Life of Patients With Atrophic and Non‐Atrophic Mandibles Using Implant‐Retained Mandibular Overdentures: 3‐Year Results of a Prospective Clinical Study,” Journal of Oral Rehabilitation 47, no. 10 (2020): 1278–1286, 10.1111/joor.13072. 32772393

[20] K. Kimoto and N. R. Garrett , “Effect of Mandibular Ridge Height on Patients' Perceptions With Mandibular Conventional and Implant‐Assisted Overdentures,” International Journal of Oral & Maxillofacial Implants 20, no. 5 (2005): 762–768. 16274151

[21] R. M. Marcello‐Machado , F. Faot , A. J. Schuster , A. M. Bielemann , G. G. Nascimento , and A. A. Del Bel Cury , “How Fast Can Treatment With Overdentures Improve the Masticatory Function and OHRQoL of Atrophic Edentulous Patients? A 1‐Year Longitudinal Clinical Study,” Clinical Oral Implants Research 29, no. 2 (2018): 215–226, 10.1111/clr.13101. 29218786

[22] C. Matthys , S. Vervaeke , W. Jacquet , and H. De Bruyn , “Impact of Crestal Bone Resorption on Quality of Life and Professional Maintenance With Conventional Dentures or Locator‐Retained Mandibular Implant Overdentures,” Journal of Prosthetic Dentistry 120, no. 6 (2018): 886–894, 10.1016/j.prosdent.2017.11.028. 29724562

[23] C. Matthys , S. Vervaeke , J. Besseler , and H. De Bruyn , “Five‐Year Study of Mandibular Overdentures on Stud Abutments: Clinical Outcome, Patient Satisfaction and Prosthetic Maintenance — Influence of Bone Resorption and Implant Position,” Clinical Oral Implants Research 30, no. 9 (2019): 940–951, 10.1111/clr.13501. 31264259

[24] S. B. Miranda , A. P. d. R. Possebon , A. J. Schuster , R. M. Marcello‐Machado , L. R. Pinto , and F. Faot , “Relationship Between Masticatory Function Impairment and Oral Health‐Related Quality of Life of Edentulous Patients: An Interventional Study,” Journal of Prosthodontics 28, no. 6 (2019): 634–642, 10.1111/jopr.13070. 31119843

[25] A. J. Schuster , R. M. Marcello‐Machado , A. M. Bielemann , A. P. d. R. Possebon , A. A. Del Bel Cury , and F. Faot , “Prosthetic Complications and Quality of Life Among Wearers of Mandibular Overdenture With the Facility‐Equator System,” Brazilian Oral Research 36 (2022): e081, 10.1590/1807-3107bor-2022.vol36.0081. 35946733

[26] A. P. d. R. Possebon , A. J. Schuster , O. L. Chagas‐Júnior , L. R. Pinto , and F. Faot , “Prosthetic Aftercare, Mastication, and Quality of Life in Mandibular Overdenture Wearers With Narrow Implants: A 3‐Year Cohort Study,” Journal of Dentistry 115 (2021): 103880, 10.1016/j.jdent.2021.103880. 34740638

[27] J. I. Cawood and R. A. Howell , “A Classification of Edentulous Jaws,” International Journal of Oral and Maxillofacial Surgery 17, no. 3 (1988): 232–236. 3139793 10.1016/s0901-5027(88)80047-x

[28] A. Leao and A. Sheiham , “Relation Between Clinical Dental Status and Subjective Impacts on Daily Living,” Community Dentistry and Oral Epidemiology 23, no. 4 (1995): 140–141. 10.1177/00220345950740071301 7560392

[29] F. A. Fontijn‐Tekamp , A. P. Slagter , A. Van der Bilt , et al., “Biting and Chewing in Overdentures, Full Dentures, and Natural Dentitions,” Journal of Dental Research 79, no. 7 (2000): 1519–1524, 10.1177/00220345000790071501. 11005738

[30] A. J. Schuster , R. M. Marcello‐Machado , A. M. Bielemann , L. R. Pinto , and F. Faot , “Is Predicting Masticatory Function Based on Mandibular Bone Atrophy as Defined by Clinical and Radiographic Parameters Possible? A Clinical Study,” Journal of Prosthetic Dentistry 121, no. 3 (2019): 432–439, 10.1016/j.prosdent.2018.05.018. 30503149

[31] L. J. da Silva , H. T. Vieira , L. L. Morel , et al., “Can Combination Syndrome Influence Functional Performance and Peri‐Implant Bone Remodeling in Mandibular Overdenture Users? Results From a 5‐Year Cohort Study,” Journal of Dentistry 160 (2025): 105878, 10.1016/j.jdent.2025.105878. 40480309

[32] K. Fueki , K. Kimoto , T. Ogawa , and N. R. Garrett , “Effect of Implant‐Supported or Retained Dentures on Masticatory Performance: A Systematic Review,” Journal of Prosthetic Dentistry 98, no. 6 (2007): 470–477, 10.1016/S0022-3913(07)60147-4. 18061741

[33] K. Kordatzis , P. S. Wright , H. J. Meijer , et al., “Posterior Mandibular Residual Ridge Resorption in Patients With Conventional Dentures and Implant Overdentures,” International Journal of Oral & Maxillofacial Implants 18, no. 3 (2003): 447–452. 12814322

[34] A. P. d. R. Possebon , A. J. Schuster , S. B. Miranda , R. M. Marcello‐Machado , O. L. Chagas‐Júnior , and F. Faot , “Do Implant‐Retained Mandibular Overdentures Maintain Radiographic, Functional, and Patient‐Centered Outcomes After 3 Years of Loading?,” Clinical Oral Implants Research 31, no. 10 (2020): 936–945, 10.1111/clr.13637. 32697874

[35] A. Van der Bilt , M. Burgers , F. M. C. Van Kampen , and M. S. Cune , “Mandibular Implant‐Supported Overdentures and Oral Function,” Clinical Oral Implants Research 21, no. 11 (2010): 1209–1213, 10.1111/j.1600-0501.2010.01915.x. 20572834

[36] W. R. Al‐Magaleh , N. A. Abbas , A. A. Amer , A. A. Abdelkader , and B. Bahgat , “Biting Force and Muscle Activity in Implant‐Supported Single Mandibular Overdentures Opposing Fixed Maxillary Dentition,” Implant Dentistry 25, no. 2 (2016): 199–203, 10.1097/ID.0000000000000374. 26684910

[37] A. J. Schuster , R. Possebon , and R. Schinestsck , “Effect of Mandibular Bone Atrophy on Maxillary and Mandibular Bone Remodeling and Quality of Life With an Implant‐Retained Mandibular Overdenture After 3 Years,” Journal of Prosthetic Dentistry 130, no. 2 (2023): 220–228, 10.1016/j.prosdent.2021.08.019. 34728072

[38] A. Kutkut , E. Bertoli , R. Frazer , G. Pinto‐Sinai , R. Fuentealba‐Hidalgo , and J. Studts , “A Systematic Review of Studies Comparing Conventional Complete Dentures and Implant Retained Overdenture,” Journal of Prosthodontic Research 62, no. 1 (2018): 1–9, 10.1016/j.jpor.2017.06.004. 28666845

[39] N. S. Abd El Rahim and A. A. Ashour , “Assessment of Quality of Life and Supporting Structures in Implant Retained Mandibular Overdenture: A 5‐Year Cohort Study,” Clinical, Cosmetic and Investigational Dentistry 14 (2022): 171–182, 10.2147/CCIDE.S364814. 35722442 PMC9198266

[40] S. A. Alfadda , N. J. Attard , and L. A. David , “Five‐Year Clinical Results of Immediately Loaded Dental Implants Using Mandibular Overdentures,” International Journal of Prosthodontics 22, no. 4 (2009): 368–374. 19639074

[41] D. R. Burns , “Mandibular Implant Overdenture Treatment: Consensus and Controversy,” Journal of Prosthodontics 9, no. 1 (2000): 37–46, 10.1053/jd.2000.6782. 11074027

[42] A. G. Payne and Y. F. Solomons , “The Prosthodontic Maintenance Requirements of Mandibular Mucosa‐ and Implant‐Supported Overdentures: A Review of the Literature,” International Journal of Prosthodontics 13, no. 3 (2000): 238–243. 11203639

