# Supplementary material for: Mandibular Atrophy and Its Impact on Overdenture Performance: Insights From a 5‐Year Longitudinal Study
Source: J Oral Rehabil. 2025 Nov 26;53(3):662–72. doi: 10.1111/joor.70113 (PMC12902194; doi:10.1111/joor.70113)
Supplement: Supplementary file 1 — Table S1: Mean ± standard deviation and median (confidence interval) of masticatory function results from Masticatory Performance (MP) and Swallowing Threshold (ST) tests (X50, B, ME 5.6 e ME2.8) according to atrophic mandible (AM) and non‐atrophic mandible (NAM) group. Table S2: Mean ± standard deviation and median (confidence interval) of the DIDL domains of subjects with atrophic (AM) and non‐atrophic mandibles (NAM) at different evaluation periods. The effect sizes (ES) of the DIDL domains at 5 years correspond to the comparison in relation to year 1. [file JOOR-53-662-s001.docx]

| **Supplementary Tables**  **Table S1.** Mean ± standard deviation and median (confidence interval) of masticatory function results from Masticatory Performance (MP) and Swallowing Threshold (ST) tests (X50, B, ME 5.6 e ME2.8) according to atrophic mandible (AM) and non-atrophic mandible (NAM) group. | | | | | | | | | | | | | | | | | | | | |  |
| --- | --- | --- | --- | --- | --- | --- | --- | --- | --- | --- | --- | --- | --- | --- | --- | --- | --- | --- | --- | --- | --- |
|  | **1 years** | |  | | **2 yeras** | |  | | **3 years** | |  | | **4 years** | |  | **5 years** | |  | |  |  |
|  | **AM (n=13)** | | **NAM (n=13)** | | **AM (n=13)** | | **NAM (n=11)** | | **AM (n=13)** | | **NAM (n=11)** | | **AM (n=12)** | | **NAM (n=10)** | **AM (n=10)** | | **NAM (n=10)** | |  |  |
| **MP** | | | | | | | | | | | | | | | | | | | |  |  |
| **MP X50** | 4.66 ± 1.15  4.50 (2.76-6.16) | | 3.85 ± 1.10  3.66 (2.47-5.83) | | 4.54 ± 1.22  4.66 (3.06-6.4) | | 3.99 ± 1.10  3.63 (2.35-5.92) | | 4.12 ± 1.39  4.46 (2.03-6.16) | | 3.99 ± 1.12  3.94 (2.05-6.17) | | 4.07 ± 1.36  4.05 (2.25-6.17) | | 3.84 ± 1.10  3.62 (2.26-6.32) | **4.65 ± 1.18**  **4.39 (2.92-6.28)** | | **4.24 ± 0.80**  **3.90 (3.42-5.90)** | |  |  |
| **MPB** | 4.25 ± 2.06  3.25 (2.22-7.85) | | 3.31 ± 1.52  2.93 (1.79-8.11) | | 3.99 ± 2.10  3.15 (2.47-9.04) | | 3.11 ± 0.71  2.83 (2.32-4.85) | | 4.18 ± 2.49  3.32 (2.24-11.12) | | 3.21 ± 0.95  2.85 (2.33-5.71) | | 3.25 ± 1.29  2.71 (2.24-6.07) | | 3.07 ± 1.52  2.73 (1.77-7.17) | 3.87 ± 1.92  2.83 (2.38-7.77) | | 2.96 ± 0.59  2.89 (2.04-4.32) | |  |  |
| **ME 5.6(%)** | 33.79 ± 23.6  27.73 (3.86-67.81) | | 17.58 ± 20.09  11.09 (0.00-59.04) | | 34.35 ± 25.88  34.78 (0.00-78.79) | | 19.39 ± 20.62  9.25 (0.01-62.18) | | 26.06 ± 26.20  18.99 (0.00-76.01) | | 20.32 ± 18.72  15.73 (2.28-69.33) | | 25.19 ± 23.83  22.48 (0.00-68.87) | | 19.71 ± 19.64  13.52 (0.00-70.81) | 34.34 ± 24.91  27.08 (4.61-73.35) | | 24.00 ± 18.60  18.41 (3.48-62.57) | |  |  |
| **ME 2.8(%)** | 18.63 ± 12.29  21.56 (2.89-36.40) | | 23.44 ± 9.84  27.4 (1.62-35.9) | | 16.21 ± 11.50  16.06 (0-34.29) | | 18.96 ± 96  21.06 (0.03-32.70) | | 19.61 ± 10.21  19.99 (0-33.64) | | 23.13 ± 7.62  21.29 (11-35.38) | | 20.81 ± 8.47  20.96 (6.95-32.47) | | 21.42 ± 8.93  19.24 (8.11- 38.34) | 19.06 ± 10.92  23.16 (1.55-33.64) | | 22.79 ± 9.16  25.05 (7.05-34.77) | |  |  |
| **ST** |  | |  | |  | |  | |  | |  | |  | |  |  | |  | |  |  |
| **ST X50** | 4.20 ± 1.32  3.82 (2.25-6.58) | 3.54 ± 1.29  3.07 (2.35-6.57) | | 3.60 ± 1.20  3.73 (1.89-5.80) | | 3.65 ± 0.85  3.45 (2.69-5.62) | | 4.00 ± 1.30  3.55 (2.22-6.24) | | 3.31 ± 1.02  2.98 (1.76-5.80) | | 3.90 ± 1.14  4.12 (2.15-5.18) | | 3.73 ± 1.02  3.67 (2.33-5.76) | | | **3.75 ± 0.63**  **3.84 (2.60-4.93)** | | **3.68 ± 0.72**  **3.42 (2.82-5.46)** | | |
| **STB** | 3.78 ± 2.07  2.97 (2.47-10.2) | 3.29 ± 2.03  2.93 (1.81-9.84) | | 3.3 ± 1.40  2.80 (2.48-7.50) | | 3.15 ± 1.11  2.75 (2.44-6.38) | | 3.55 ± 1.63  2.88 (2.06-6.91) | | 2.92 ± 0.76  2.81 (2.11-4.87) | | 2.69 ± 0.46  2.62 (2.14-3.72) | | 3.16 ± 1.61  2.59 (2.28-7.65) | | | 2.52 ± 0.29  2.55 (2.12-3.00) | | 3.01 ± 0.83  2.73 (2.49-5.28) | | |
| **ME 5.6(%)** | 26.0 ± 25.3  16.66 (0-79.96) | 17.16 ± 25.61  6.12 (0-85.20) | | 15.10 ± 19.30  11.48 (0-55.43) | | 12.47 ± 14.73  5.89 (1.73-48.30) | | 21.00 ± 22.10  11.73 (0-71.24) | | 16.07 ± 19.98  8.34 (0-54.74) | | 18.19 ± 17.70  16.12 (0-43.79) | | 16.81 ± 17.13  15.19 (0-54.56) | | | **17.50 ± 10.22**  **15.71 (0-41.70)** | | **12.21 ± 13.35**  **8.32 (0-44.07)** | | |
| **ME 2.8(%)** | 23.2 ± 12.9  22.14 (2.56-52.33) | 23.2 ± 9.32  25.28 (1.63-36.08) | | 12.2 ± 13.20  5.34 (0.01-31.72) | | 13.27 ± 14.77  2.31 (0.15-40.70) | | 21.30 ± 12.50  23.99 (0-35.43) | | 25.64 ± 11.62  29.70 (3.73-42.02) | | 19.94 ± 4.99  19.76 (12.74-29.32) | | 23.13 ± 10.33  24.03 (1.63-35.92) | | | 25.28 ± 6.59  25.47 (14.40-37.43) | | 27.29 ± 7.94  28.01 (7.85-35.24) | | |
| **Time (s)** | 60.2 ± 33.1  47.53 (23.49-145.30) | 52.58 ± 16.74  51.35 (31.49-78.06) | | 64.40 ± 23.40  59.84 (36.15-114.27) | | 46.69 ± 12.67  45.58 (29.09-65.24) | | 53.60 ± 24.60  46.38 (29.37-111.95) | | 54.12 ± 12.75  53.75 (31.60-71.06) | | 54.95 ± 19.11  52.00 (26.50-87.00) | | 44.32 ± 14.73  41.00 (26.00-77.00) | | | 57.86 ± 18.35  52.64 (39.00-93.74) | | 51.59 ± 18.33  46.84 (28.50-86.06) | | |
| **N° of Cycles** | 58.5 ± 27.9  47 (37-134) | 55.62 ± 14.91  50 (38-78) | | 67.40 ± 23.70  62 (36-112) | | 51.27 ± 11.63  51 (35-70) | | 58.90 ± 28.20  52 (33-117) | | 56.13 ± 24.48  60 (0.41-81) | | 61.20 ± 18.53  61.00 (35.00-87.00) | | 53.20 ± 20.53  49.00 (30.00-107.00) | | | 69.30 ± 23.76  65.50 (48.00-130.00) | | 59.60 ± 22.36  59.50 (28.00-100.00) | | |

**Table S2**. Mean ± standard deviation and median (confidence interval) of the DIDL domains of subjects with atrophic (AM) and non-atrophic mandibles (NAM) at different evaluation periods. The effect sizes (ES) of the DIDL domains at 5 y correspond to the comparison in relation to year 1.

|  | **1 year** | | **2 years** | | **3 years** | | **4 years** | | **5 years** | | **EFFECT SIZE (5-1)** | |
| --- | --- | --- | --- | --- | --- | --- | --- | --- | --- | --- | --- | --- |
|  | **AM (n=13)** | **NAM (n=13)** | **AM (n=13)** | **NAM (n=11)** | **AT(n=13)** | **NAM (n=11)** | **AT(n=12)** | **NAM (n=10)** | **AM (n=10)** | **NAM (n=10)** | **AM** | **NAM** |
| **Appearance** | 1 ± 0  (1-1) | 1 ± 0  (1-1) | 1 ± 0  (1-1) | 0.95 ± 0.15  (0.5-1) | 0.92 ± 0.28  (0-1) | 0.75 ± 0.60  (−1-1) | 0.77 ± 0.44  (0-1) | 0.85 ± 0.47  (-0.5-1) | 0.75 ± 0.43  (0-1) | 0.88 ± 0.27  (0.25-1) | 0.0 | 0.0 |
| **Pain** | 0.87 ± 0.22  (0.5-1) | 0.85 ± 0.24  (0.5-1) | 1 ± 0  (1-1) | 0.91 ± 0.20  (0.5-1) | 0.92 ± 0.16  (0.5-1) | 0.91 ± 0.17  (0.5-1) | 0.64 ± 0.46  (0.5-1) | 0.78 ± 0.34  (0-1) | 0.43 ± 0.55  (-0.75-1) | 0.70 ± 0.48  (-0.5-1) | 2.0 | 1.3 |
| **Oral Confort** | 0.78 ± 0.31  (0.14-1) | 0.82 ± 0.32  (−0.14-1) | 0.84 ± 0.22  (0.43-1) | 0.91 ± 0.16  (0.57-1) | 0.88 ± 0.13  (0.71-1) | 0.87 ± 0.16  (0.57-1) | 0.46 ± 0.66  (-1-1) | 0.87 ± 0.32  (0-1) | 0.09 ± 0.62  (-1-1) | 0.43 ± 0.46  (0-1) | 2.2 | 1.5 |
| **General Performance** | 0.98 ± 0.07  (0.73-1) | 0.97 ± 0.08  (0.73-1) | 1 ± 0  (1-1) | 0.98 ± 0.06  (0.8-1) | 0.96 ± 0.09  (0.71-1) | 0.92 ± 0.20  (0.33-1) | 0.75 ± 0.43  (0.73-1) | 0.98 ± 0.06  (0-1) | 0.69 ± 0.41  (0.6-1) | 0.86 ± 0.12  (0.73-1) | 3.8 | 3.8 |
| **Eating/Chewing** | 1 ± 0  (1-1) | 0.85 ± 0.55  (−1-1) | 1 ± 0  (1-1) | 0.95 ± 0.15  (0.5-1) | 0.90 ± 0.22  (0.33-1) | 0.74 ± 0.38  (0-1) | 0.74 ± 0.43  (0.67-1) | 0.67 ± 0.57  (-0.33-1) | 0.35 ± 0.72  (-1-1) | 0.73 ± 0.47  (-0.33-1) | 0.0 | 0.5 |
